# Vascular leakage during circulatory failure: physiopathology, impact and treatments

**DOI:** 10.1186/s13613-025-01474-8

**Published:** 2025-06-06

**Authors:** Jérémie Joffre, Peter Radermacher, Hatem Kallel, Iris Marangon, Alexandre Rutault, Yaël Levy, Alexandre Gaudet, Benjamine Sarton, Louis Kreitmann, Lucillia Bezu, Meryl Vedrenne, Thomas Maldiney, Youenn Jouan, Sarah Benghanem, Laure Stiel, Stéphane Germain, Nicolas Bréchot, For the ‘commission recherche translationnelle de la société de réanimation de langue française’

**Affiliations:** 1 Service de Réanimation Médicale, Hôpital Saint Antoine, Assistance Publique-Hôpitaux de Paris (AP-HP), Paris, France; 2 Centre de Recherche Saint Antoine INSERM, U938, Sorbonne University, Paris, France; 3 Institute for Anesthesiological Pathophysiology and Process Engineering, Ulm University, 89081 Ulm, Germany; 4 Service de Réanimation, Centre Hospitalier de Cayenne, Guyane, France; 5 Center for Interdisciplinary Research in Biology, Collège de France, Centre National de la Recherche Scientifique, Institut National de la Santé et de la Recherche Médicale (INSERM), Université PSL, Paris, France; 6 Pediatric and Neonatal Intensive Care Unit, Armand-Trousseau Hospital, APHP, 26 Avenue du Dr Arnold Netter, 75012 Paris, France; 7 Sorbonne University, Paris, France; 8 INSERM, IMRB, Univ Paris Est Créteil, 94010 Créteil, France; 9 Department of Intensive Care Medicine, Critical Care Center, CHU Lille, Univ. Lille, 59000 Lille, France; 10 CIIL (Centre d’Infection et d’Immunié de Lille), Institut Pasteur de Lille, U1019-UMR9017, 59000 Lille, France; 11 Service de Réanimation Polyvalente Purpan, Centre Hospitalier Universitaire de Toulouse, Toulouse, France; 12 ToNIC Lab (Toulouse NeuroImaging Center) INSERM/UPS UMR 1214, 31300 Toulouse, France; 13 Centre for Antimicrobial Optimisation, Department of Infectious Disease, Faculty of Medicine, Imperial College London, London, W12 0HS UK; 14 Department of Critical Care Medicine, Imperial College Healthcare NHS Trust, London, UK; 15 Département d’Anesthésie, Chirurgie, Interventionnel et Service de Réanimation, Gustave Roussy, FR94805 Villejuif, France; 16 U1138 Metabolomics and Cell Biology Platform, Université Paris Saclay, 94805 Villejuif, France; 17 Service de Réanimation Médicochirurgicale Pédiatrique, CHU Necker-Enfants Malades, Assistance Publique-Hôpitaux de Paris (AP-HP), Paris, France; 18 Unité de VNI Et du Sommeil de l’enfant, URP7330 VIFASOM, Université Paris Cité, Paris, France; 19 Department of Intensive Care Medicine, William Morey General Hospital, Chalon-Sur-Saône, France; 20 Lipness Team, Institut National de La Santé et de la Recherche Médicale (INSERM) Research Centre Lipides, Nutrition, Cancer - Unité Mixte de Recherche (LNC-UMR)1231, University of Burgundy, Dijon, France; 21 Service de Médecine Intensive Réanimation, CHRU Tours, Tours, France; 22 Services de Réanimation Chirurgicale Cardiovasculaire Et de Chirurgie Cardiaque, CHRU Tours, Tours, France; 23 Faculté de Médecine de Tours, INSERM, U1100 Centre d’Etudes des Pathologies Respiratoires, Tours, France; 24 Service de Médecine Intensive Réanimation, Hôpital Cochin, Assistance Publique-Hôpitaux de Paris (AP-HP), Paris, France; 25 Université Paris Cité, Paris, France; 26 Department of Intensive Care Medicine, Groupe Hospitalier de la Région Mulhouse Sud Alsace, Mulhouse, France; 27 Lipness Team, INSERM Research Team, LNC, UMR 1231 and LabEx LipSTIC, University of Burgundy, Dijon, France; 28 Service de Réanimation Médicale, Hôpital Européen Georges Pompidou, Assistance Publique-Hôpitaux de Paris (AP-HP), Paris, France; 29 Département de médecine intensive - réanimation et médecine hyperbare, Centre Hospitalier Universitaire, 4 rue Larrey, 49933 Angers Cedex 09, France

**Keywords:** Vascular leakage, Shock, Circulatory failure, Systemic inflammation, Review, Endothelium, Sepsis, Cardiogenic shock, Hemorrhagic shock

## Abstract

Vascular leakage has emerged as a major factor during circulatory failure. Triggered by the inflammatory process following the recognition of both pathogen-associated molecular patterns (PAMPs) and damage-associated molecular patterns (DAMPs), it worsens circulatory failure through the hypovolemia it induces. It may also crucially participate in secondary microcirculation disorders and organ dysfunctions, due to interstitial edema resulting from extravascular fluid accumulation. Accordingly, fluid balance, i.e., the difference between fluid intake and output, is directly related with outcomes during the different types of shock. Moreover, controlling vascular leakage had beneficial effects in various animal models of circulatory failure. However, despite promising preclinical findings, no routine drug is currently available to control vascular leakage in humans. This review depicts the mechanisms involved in the maintenance of a quiescent endothelium and those implicated in the destabilization of its barrier function in various forms of shocks. It further describes available tools to explore vascular leakage and the most advanced treatments under development.

## Introduction: vascular leakage, a key player during circulatory failure

The endothelium, which covers a surface area of more than 1000 m^2^ in the human body, has a key role in regulating leucocyte trafficking and tissue homeostasis [1]. While a *localized* increase in permeability is essential for the healing process following tissue injury, the systemic spread of hyperpermeability may become highly deleterious. This *"vascular leakage"* (or *"capillary leakage"*) is induced by systemic inflammation associated with circulatory shock and represents an important feature of circulatory failure. Vascular leakage worsens circulatory failure by inducing hypovolemia and may promote organ dysfunction through the interstitial edema it induces [2–4]. Various experimental studies demonstrated that even small increases in the water content of organs may crucially affect their function [5]. Vascular leakage also decreases hydrostatic and osmotic pressures in small vessels compared to the adjacent interstitium, which is thought to induce their occlusion and contribute to the microcirculation disorders observed during shock states [6]. Although it has been initially described during sepsis, inflammation-induced vascular leakage is also a hallmark of *"sterile"* forms of shock, e.g., cardiogenic shock [7, 8], post-resuscitation syndrome [9, 10], and resuscitation from hemorrhagic shock [11]. Accordingly, fluid balance is an independent predictor of mortality during septic or cardiogenic shock [8, 12–14]. In turn, controlling capillary leakage was beneficial during experimental circulatory failure [15–17]. However, the pathophysiology of vascular leakage remains poorly described in humans, and no routine therapeutic is currently available to control it [4].

**Fig. 1:**
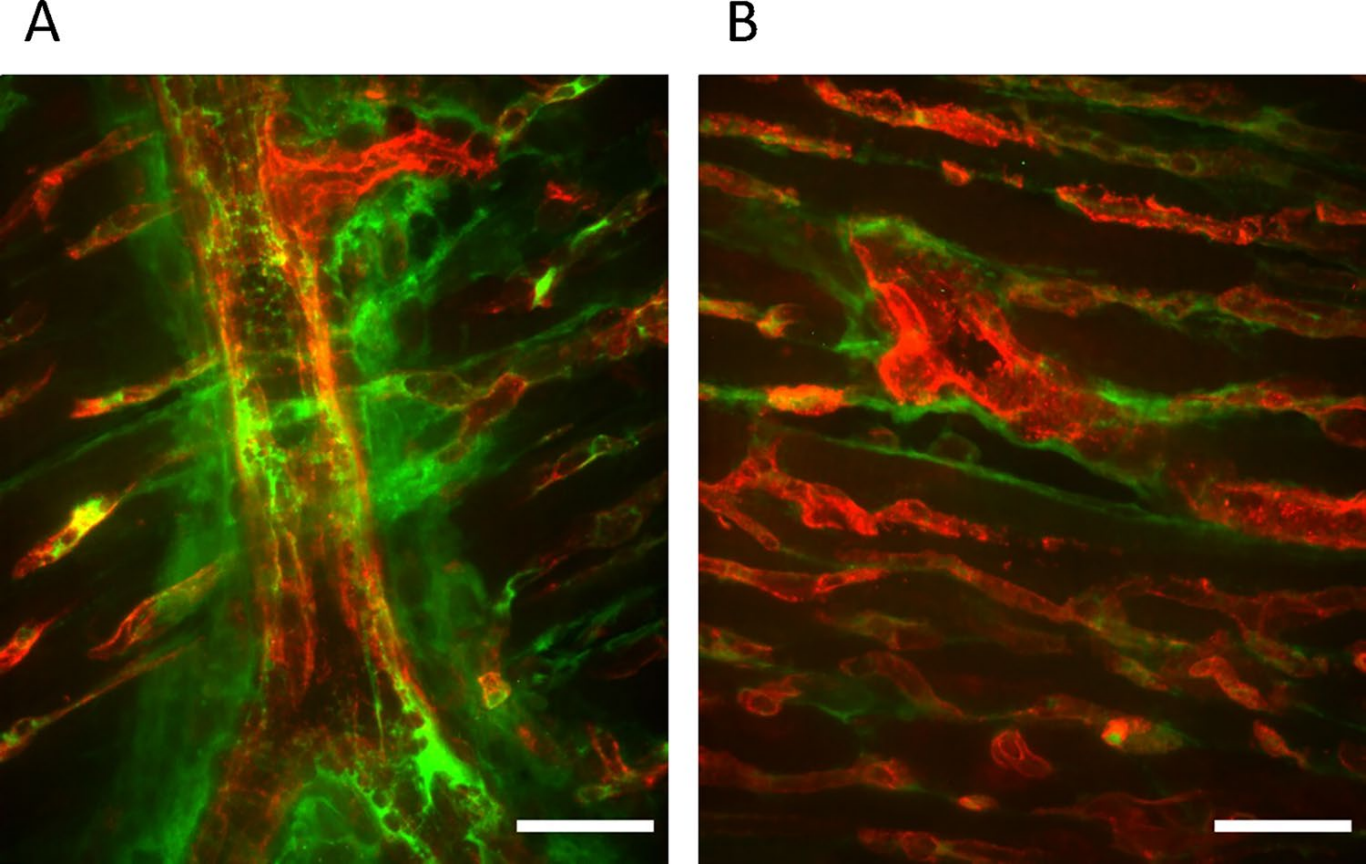
**A** Representative heart cross sections from mice injected i.v. with fluorescent 70-kDa dextran (green) at the time of resuscitation (Low-Flow = 0 min) and harvested at Low-Flow min 4, in a model of resuscitated cardiac arrest, showing a massive extravasation of the dye. **B** sham animals (without cardiac arrest) in the same model. Vessels were counter-stained with fluorescent isolectin (red). Scale bar = 25 µm

Many techniques have been developed to quantify the degree of leakage in animal models. The organ total/dry weight ratio (before and after dehydration) allows for a rough assessment of fluid accumulation over time [18]. Other authors utilized the extravasation of various dyes injected intravenously, quantifying the leakage through histology or measuring their elution from organs. These dyes enable a more precise quantification of the degree of leakage within shorter time windows, the most commonly used ones being Evans blue [19], fluorescent dextrans, and microspheres [20] (Fig. 1).

Only limited tools are available in humans. Transpulmonary thermodilution is a routinely available method. Still, it only explores the leakage from the *perfused* pulmonary vascular bed based on the extravascular lung water content and the pulmonary vascular permeability index [21–23]. Other methods of interest use indocyanine green, which rapidly binds to circulating proteins when injected intravenously. However, as multiple factors affect its clearance (e.g., vascular permeability, cardiac output, and liver function), its plasma concentration can only reliably estimate plasma volume [24–26]. Indexing plasma volume evaluated by indocyanine green on the initial distribution volume of glucose (after a simultaneous injection of glucose, distributing rapidly in the extracellular space), may allow to more specifically address vascular permeability to proteins [24, 25]. However, this technique remains technically demanding and requires further validation. Bioelectrical impedance, which measures the water content between two electrodes, also yielded promising preliminary results [26]. Nevertheless, fluid balance remains the predominant marker of leakage in the clinical setting, although it is influenced by several confounding factors, e.g., local fluid loading policies, the individual patient's proteinemia, vasoplegia, and renal function [10, 14–16].

## Pathophysiology of vascular leakage during sepsis

### Endothelial cell quiescence and barrier function

**Fig. 2:**
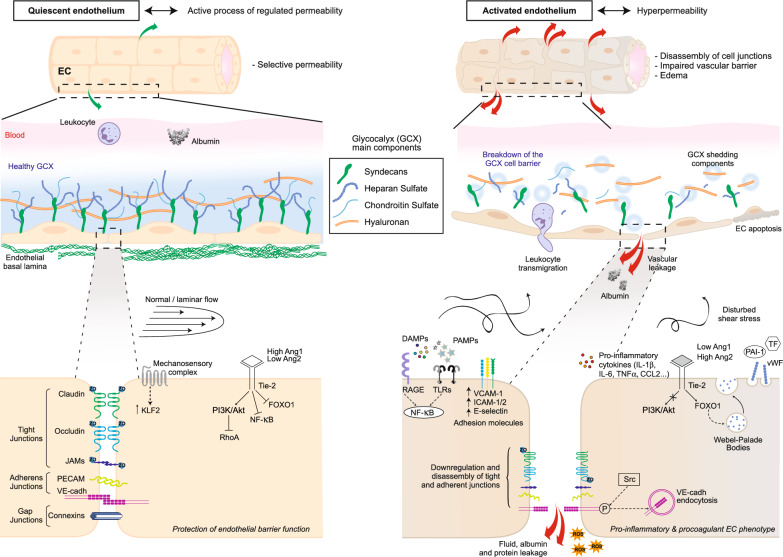
Schematic representation of mechanisms implicated in endothelial barrier function and vascular hyperpermeability during circulatory failure

The barrier function of the quiescent endothelium allows a tight regulation of tissue homeostasis. Maintenance of the endothelium in a quiescent state is an active process involving a combination of many signaling pathways (Fig. 2) [27]. Laterally, endothelial cells (EC) interact strongly with adjacent EC via inter-endothelial junctions [28], which regulate endothelium integrity and paracellular trafficking of fluids and inflammatory cells [29, 30]. Three types of junctions are involved in EC-EC adhesion:

- *Gap junctions*, which rely on proteins from the connexin family, serve as key regulators of small molecules trafficking across the endothelium [31].
- *Tight junctions* are transmembrane proteins that interact with adjacent endothelial cells [32]. They are composed of members of claudin, occludin, junction-associated molecule (ESAM) and junctional adhesion molecules (JAMs), which bind to actin cytoskeleton through zona occludens 1 protein (ZO-1), cingulin, and paracingulin.
- *Adherens junctions* involve homophilic interactions of vascular endothelial (VE)-cadherin [33, 34].

VE-cadherin is a crucial regulator of paracellular permeability to fluids, which allows the trafficking of small molecules (< 3.6 μm, < 40 kDa) at basal state. Accordingly, an anti-VE-cadherin antibody induced major vascular leakage in mice in vivo [35].

Signals from the basement membrane also contribute to the maintenance of the quiescent state. In particular, interactions between integrins and extracellular matrix components activate downstream intracellular pathways responsible for stabilizing the endothelial barrier, such as the PI_3_K/AKT pathway [36]. Matrikines (i.e., growth factors embedded in the extracellular matrix) and matricellular proteins also contribute to signaling through a quiescent state. Co-stimulation of vascular endothelial growth factor-A (VEGF-A) and fibroblast growth factors (FGF), particularly, is a strong signal for survival and maintenance of a quiescent phenotype through ERK1/2 signaling [27, 37].

The glycocalyx has also been referred to assume a significant role for maintaining endothelial barrier integrity. Composed of a complex network of glycoproteins and membrane-bound proteoglycans, it acts as a protective “jelly-like” layer coating the surface of vascular EC (Fig. 2). Maintaining proteins at the center of the vessel, it creates an osmotic gradient preventing water from leaking through the endothelial barrier. In addition, the glycocalyx also has essential signaling functions, particularly concerning flow sensing and its downstream mechano-transduction [38]. Glycocalyx components such as Syndecan-4 associating with β1 integrin [36], function as receptors for fibroblast growth factors (FGFs), vascular endothelial growth factors (VEGFs), and platelet-derived growth factors (PDGFs) [39], to enhance EC barrier function.

Finally, shear stress is an important signal that promotes vascular integrity and the quiescence of EC [40]. The expression of the Krüppel-like family of transcription factors KLF2/4 is induced by laminar shear stress and maintains a quiescent endothelium by downregulating inflammation and inhibiting the pro-coagulant activity of EC. This consists in decreasing the expression of the surface tissue factor and PAI-1, and increasing thrombomodulin expression [37, 41]. Platelet endothelial cell adhesion molecule-1 (PECAM-1), VE-cadherin, and VEGF receptor-2 (VEGFR2) also form an essential mechanosensory complex mediating endothelial cell response to shear stress [27, 42, 43]. Circulating factors are also involved in maintaining a quiescent phenotype. In particular, Angiopoietin-1, secreted by adjacent mural cells, signals through its endothelial receptor Tie2 and activates the PI_3_K/AKT pathway [44].

### Microvascular endothelial activation and sepsis-induced capillary leak

In acute inflammation, both pathogen-associated molecular patterns (PAMPs) and damage-associated molecular patterns (DAMPs) activate multiple redundant and pleiotropic pathways in microvascular endothelial cells [6]. This initiates a shift from a homeostatic phenotype characterized by hyperselective and regulated permeability to an activated phenotype characterized by hyperpermeability [45, 46]. Several mechanisms contribute to inflammation-induced permeability: destabilization of adherens and tight junctions, glycocalyx breakdown, oxidative stress, and cell death (Fig. 2).

The integrity of adherens junctions is modulated by tyrosine phosphorylation of its components, particularly VE-cadherin [47]. VE-cadherin is phosphorylated by stimuli inducing vascular leakage, mostly depending on Src and RhoA kinase activity, leading to its endocytosis and adherens junction destabilization. Phosphorylated RhoA and Rock also cause the formation of actin stress fibers, generating forces at the lateral cellular wall, which, in turn, widens the intercellular space and participates in junction disassembly and increased permeability [30, 48].

Consequently, various mediators such as Toll-Like Receptor (TLR) agonists, interleukin-6 (IL-6), thrombin, or VEGF may increase VE-cadherin phosphorylation, leading to its endocytosis in clathrin-coated vesicles [49]. For example, VEGF initiates a signaling cascade involving Src kinase, Vav2, and Rac, which triggers the serine phosphorylation of VE-cadherin. This results in the recruitment of β-arrestin2 and culminates in the endocytosis of VE-cadherin [50]. Inhibiting this VEGF signaling pathway is one of the mechanisms by which the vascular protective agent angiopoietin-1 (Ang1) prevents VE-cadherin internalization and vascular leakage [51]. Additionally, cytokine-induced phosphorylation of the VE-cadherin cytoplasmic domain has been reported to trigger the cleavage of its extracellular domain [52], producing a soluble form detectable in the plasma as a biomarker of endothelial activation/dysfunction [53, 54]. Recently, Yang et al*.* have demonstrated that, during sepsis, circulating lactate activates the calpain1/2 in an ERK-dependent pathway, provoking the VE-cadherin proteolytic cleavage and its endocytosis in EC. This study also demonstrated that both genetically or pharmacologically inhibiting lactate signaling alleviated sepsis-associated capillary leak and improved outcomes in murine polymicrobial sepsis, suggesting a direct role of lactate in promoting endothelial permeability [55]. Similar to adherens junctions, several studies have reported the disruption of endothelial tight junctions in sepsis, reducing the protein levels of occludin and zonula occludens: for instance, tumor necrosis factor-alpha (TNF-α) was shown to disrupt claudin-5 at endothelial cell–cell junctions through NF-κB pathway activation [56].

In addition to cell–cell junctions, glycocalyx degradation can promote microvascular permeability and a local increase in oxidative stress [57]. Human studies provided evidence that sepsis and other acute inflammatory illnesses are associated with shedding of the glycocalyx, as measured by syndecan-1, hyaluronan, or glycosaminoglycans [58–61]. Their circulating levels are related to capillary leakage and poor outcome [62–65]. In a lipopolysaccharide (LPS) model of endotoxemia in mice, Kataoka et al*.* demonstrated that glycocalyx destruction increased microvascular macromolecule permeability and leukocyte adhesion [66]. Moreover, the intact glycocalyx serves as a sanctuary for two major antioxidant enzymes: superoxide dismutase (SOD) [67] and activated xanthine oxidase [68]. Therefore, glycocalyx degradation may cause a shift towards a pro-oxidant state. In turn, reactive oxygen species (ROS) can amplify glycocalyx degradation via proteolytic cleavage of syndecan-1 and sulfated glycosaminoglycan, ultimately further aggravating endothelial permeability [69, 70].

Finally, redox imbalance can induce EC death through apoptosis or necrosis. ROS (e.g., H_2_O_2_, OH^•^¯) and reactive nitrogen species (e.g., ONOO^−^) trigger cell death programs both via the mitochondrial and extrinsic (death receptor and endoplasmic reticulum) pathways [71, 72]. The degree of redox imbalance determines whether EC undergo apoptosis or necroptosis, both of which can significantly enhance endothelial dysfunction [73]. Endothelial cell death involves the loss of structural and membrane properties, thus exacerbating permeability, capillary protein and fluid leakage, and, consequently, organ injury. In a model of peritonitis in mice, Gill et al*.* identified apoptotic microvascular endothelial cells in the lung and reported that pretreatment with a pan-caspase inhibitor attenuated capillary leakage [74]. This suggests that endothelial cell apoptosis may also mediate lung vascular hyperpermeability. Finally, necrotic cells release large amounts of DAMPs and phosphatidylserine (PS)-positive microparticles, amplifying local inflammation and enhancing capillary leakage [75]. Beyond apoptosis and necrosis, other forms of endothelial cell death have been shown to be involved in sepsis. “Pyroptosis” is a programmed form of necrosis activated by intracellular sensors of microbial products, which, in turn, trigger the formation of an intracellular complex known as the “inflammasome”. This process amplifies the release of pro-inflammatory cytokines and causes damage to the endothelium. Caspase-1, a key effector of pyroptosis, has been shown to contribute to sepsis lethality [76]. More recently, PANoptosis has been described as a form of cell death that integrates features of pyroptosis, apoptosis, and necroptosis, with emerging evidence supporting its role in sepsis [77, 78]. However, the relative contribution of these forms of cell death to vascular leakage remains to be determined.

It could be argued that controlling vascular leakage may impair the immune response to pathogens during sepsis. However, numerous studies using intravital microscopy as well as cell and molecular biology have challenged the notion that vascular leakage is necessary for leukocyte trafficking through the endothelial barrier. In fact, an adequate and competent immune response involves tight regulation of leukocyte trafficking, which relies on a controlled “unzipping” of endothelial cell–cell junctions [79, 80]. Accordingly, several agents have been shown to reduce vascular leakage in animal models of sepsis without impairing leukocyte recruitment or pathogen clearance [79]. This “uncoupling” of fluid and leukocyte extravasation further reinforces the rationale for controlling trans-endothelial leakage during sepsis.

## Vascular leakage in other forms of circulatory failure

### Post-resuscitation syndrome

Vascular leakage is also a hallmark of the post-resuscitation syndrome [81], which shares many pathophysiological features with sepsis [9]. Ischemia–reperfusion (I/R) injury after cardiac arrest triggers an intense inflammatory reaction, with levels of circulating cytokines comparable with sepsis [82, 83], and high amounts of fluids needed to correct hypovolemia [9, 10, 84]. The resulting interstitial edema is thought to assume significant importance for organ function: an only 4% increase in myocardial water content was sufficient to reduce cardiac function by more than 50% in animals [5, 85]. Myocardial edema, which occurs within minutes, might, therefore, contribute to the transient myocardial dysfunction observed after cardiac arrest [84]. Likewise, both in animal and human studies, post-cardiac arrest neurological deficit was directly related to cerebral edema [86, 87]. Targeting vascular leakage, hence, is a promising therapeutic approach after cardiac arrest.

During ischemia, the inflammatory process is induced by the release of DAMPS, mainly consisting of damaged cell debris released after necrosis (membrane, DNA, histone fragments…) [88]. DAMPs are recognized as danger signals by Pattern-Recognition Receptors (PRRs) expressed by resident inflammatory cells (macrophages and mastocytes), initiating an inflammatory response [89] and activating EC by producing pro-inflammatory cytokines [89–91]. Among these DAMPs, High-Mobility Group Box 1 (HMGB1) and S100 A8/A9 are pivotal during ischemia [92, 93], by activating the nuclear transcription factor NF-κB that signals through toll-like receptors 2 and 4 (TLR2/4) and receptors for advanced glycation end-products (RAGE), expressed by inflammatory cells. EC activation is responsible for the recruitment of inflammatory cells, which further enhances the inflammatory response to ischemia through ROS generation [89, 94]. The resulting inflammation strongly impairs the stability of inter-endothelial cell junctions. Upon activation, EC release the contents of Weibel-Palade bodies into the circulation, which contain a wide range of molecules responsible for endothelium activation and destabilization. Among those, Angiopoietin-2 is a potent inducer of vascular permeability as a competitor agonist of Angiopoietin-1 on the Tie-2 receptor [95]. Von Willebrand Factor, in combination with the release of tissue factor (TF) and plasminogen activator inhibitor-1 (PAI-1) by activated EC, induces a procoagulant endothelial state [43, 91] and thrombin generation, which contributes to vascular leakage from ischemic vessels [30]. ROS, along with many substances released by inflammatory cells (histamine, bradykinin, nitric oxide, leukotrienes, thromboxanes and prostaglandins), further increase vascular hyperpermeability [30, 48, 96, 97]. Finally, as for sepsis, the glycocalyx undergoes significant degradations during ischemia, contributing to capillary leakage [98].

In conclusion, while vascular leakage exhibits common pathophysiological features both during sepsis and the post-resuscitation syndrome, the precise common mechanisms, however, and any differences remain to be better described. Likewise, the contribution of tissue ischemia to sepsis-induced vascular leakage must be better characterized.

### Cardiogenic shock

Severe cardiogenic shock is also responsible for ischemia-induced tissue lesions, thus triggering systemic hyper-inflammation. This leads to the induction of a complex hemodynamic profile, combining features of both low cardiac output and inflammation-induced vasoplegia, and vascular leakage [99], the degree of which is directly related to patient severity [8]. Accordingly, the angio-1/2 ratio correlates with mortality [7]. Nevertheless, controlling vascular leakage during cardiogenic shock remains an unresolved issue.

### Hemorrhagic shock

Similar to sepsis, trauma and hemorrhage lead to systemic hyper-inflammation triggered by direct tissue damage per se [100, 101] and shock-induced tissue hypoxia [102]. Therefore, “repayment of the O_2_ debt” [103] to restore/maintain tissue O_2_ supply is a cornerstone of the management. However, in analogy to the post-resuscitation syndrome, restoring tissue perfusion induces an I/R-type of injury [104]. The type and amount of aggressive fluid resuscitation [105, 106] and catecholamine infusion [107] to achieve adequate perfusion, may further aggravate this effect. Hence, diffuse capillary leakage was already described in the context of trauma-and-hemorrhage in the 1970s. However, despite progress in the understanding of its pathophysiology, its precise mechanisms remain unclear [108], and, in particular, practical measures for its prevention and/or treatment are still lacking. The local trauma-induced release of cytokines, complement, arachidonic acid derivatives, and ROS primarily induces repair processes. However, an overwhelming injury may cause systemic spillover of these mediators, thereby initiating the above-mentioned systemic hyper-inflammation. In addition, whereas a single, moderate, and/or localized trauma does not per se cause this systemic response, any additional, even delayed, second stress may do so [109]. This is referred to as the"two-hit"theory [109, 110], emphasizing the role of impaired handling of a second stress, e.g., follow-up surgery, secondary infection, and/or transfusion of blood products.

Evidence of trauma-related vascular leakage has been reported for virtually all organs, i.e., the lung [111–117], the gut as documented by translocation of endotoxin and/or live bacteria [116, 118–122], the heart [123, 124], the liver [115, 125], the kidney [114, 115, 126, 127] and the brain [128]. Not only does trauma aggravate vascular barrier dysfunction resulting from hemorrhagic shock [129], but also conversely hemorrhage aggravates any trauma-related barrier destabilization [115, 116]. In this context, hemorrhage increased syndecan-1 levels beyond those induced by trauma alone, suggesting that the endothelial glycocalyx may assume importance for hemorrhage-related vascular leakage. In fact, exogenous albumin attenuated albumin leakage into the extravascular space, indicating the role of the endothelial glycocalyx for intravascular maintenance of plasma proteins [130].

In experimental trauma-and-hemorrhage and subsequent (fluid) resuscitation, various therapeutic strategies have been tested to reduce microvascular leakage (for a systematic review see [131]): these approaches comprise modulation of energy metabolism (e.g. hypothermia [126, 132]), targeting inflammatory pathways (e.g., via complement blockade [133] or vagus nerve stimulation [134]), the angiopoietin/Tie-2 system (e.g., vasculotide [135]), administration of sex hormones [136, 137] or sphingosine [138], the use of hyperoxia (i.e., increased inspired O_2_ fractions [126, 127]), vasopressin [139] or its analogues [140] rather than catecholamines to counteract hypotension, and targeted choice of intravascular fluid resuscitation (e.g., the use of blood products [141] and/or synthetic colloids rather than crystalloids [142], or hypertonic saline [143]; for detailed review see [144, 145]).

Clearly, the existing data remains equivocal, and it remains an open question whether any promising experimental finding can be confirmed in clinically relevant resuscitated models of trauma and hemorrhage. So far, however, none of these therapeutic strategies has found its way into clinical practice.

## More advanced therapeutic strategies under investigation to control vascular leakage during circulatory failure

A wide range of potential treatments to prevent or reduce vascular leakage during the various forms of circulatory failure have been or are currently being investigated in pre-clinical studies [146]. Some are more advanced and have translated to clinical investigations.

### Vasopressors and endothelial permeability

Vasopressors are pivotal during the early management of shock to restore mean arterial pressure and sustain organ perfusion. Nevertheless, their microvascular effects have often been overlooked, particularly regarding endothelial permeability. In an era marked by increasing interest in non-catecholaminergic agents like vasopressin or angiotensin-II, the examination of microvascular effects of both catecholaminergic and non-catecholaminergic agents becomes imperative.

In ex vivo experiments utilizing human lung microvascular endothelial cells (L-HMVEC), Joffre et al. reported that epinephrine and norepinephrine significantly reduce Toll-Like Receptor (TLR) agonist and proinflammatory cytokine-induced endothelial permeability [147]. This effect was demonstrated to be mediated through both ß1 and ß2-adrenergic receptors. Mechanistically, ß2-adrenergic receptor activation reduces LPS-induced permeability through cytoskeletal rearrangement, contributing to the maintenance of impermeability under inflammatory conditions [148, 149]. Clinical trials also support these experimental findings; in the BALTI-1 study on ARDS patients, the salbutamol group exhibited significantly lower lung water at day 7 compared to the control group [150], suggesting that ß-adrenergic stimulation may attenuate capillary leakage. This effect, however, did not translate into increased survival in the larger confirmatory randomized trial BALTI-2 [151]. The CENSER study on septic shock patients receiving early norepinephrine indicated a lower occurrence of pulmonary edema (14% vs. 28%; p = 0.004) despite similar fluid volumes, suggesting that early catecholamine administration may limit sepsis-induced capillary leak syndrome [152].

Regarding vasopressin, in vivo experiments of pneumonia-induced sepsis in sheep demonstrated that the V2-receptor antagonist tolvaptan significantly attenuated fluid retention and reduced lung water content [153]. However, in rats undergoing hemorrhagic shock, vasopressin worsened pulmonary and renal capillary leakage [139]. Vasopressin receptor blockade reversed blood–brain barrier hyperpermeability during experimental autoimmune encephalomyelitis in rats, suggesting that vasopressin might be deleterious to blood–brain barrier permeability [154]. Therefore, arginine vasopressin could potentially exacerbate capillary leak.

Data regarding angiotensin-2 are limited. Ex vivo studies using transcriptomics reported that angiotensin-2 induced a concentration and time-dependent increase in VEGF mRNA expression by human vascular smooth muscle cells [155]. In human umbilical vein endothelial cells (HUVEC), angiotensin II was reported to increase permeability and plasmalemmal vesicle-1 (PV-1) expression, a protein associated with microvascular leakage [156]. Similarly, under high glucose concentrations, angiotensin II also aggravated LPS-induced permeability in L-HMVEC [157]. Nevertheless, using experimental transendothelial hydraulic permeability measurements, Victortino et al*.* observed that while microvascular permeability is increased by angiotensin-2 under basal conditions, it might be reduced in ATP-activated endothelium [158]. Overall, data on microvascular function and capillary leak are crucially needed from clinical trials exploring the effects of non-catecholaminergic vasopressors.

#### Adrecizumab

Adrenomedullin is a protein belonging to the calcitonin gene-related family that has dual vascular effects. It reduces inflammation-induced endothelial hyperpermeability and promotes smooth muscle cell-related vasorelaxation [159–162]. Adrecizumab, a humanized monoclonal non-neutralizing anti-adrenomedullin antibody (clone HAM8101), increased adrenomedullin bio-availability in the plasma by preventing adrenomedullin degradation and inhibiting its translation from the plasma to the interstitium. Therefore, it was tested during sepsis to attenuate both vascular leakage and vasoplegia [159]. It efficiently reduced vascular leakage and improved survival in pre-clinical models of sepsis [163], and, consequently, was evaluated in a phase 2a double-blind randomized trial, the AdrenOSS-2 biomarker-guided trial [159]. 301 septic shock patients with plasma bio-adrenomedullin concentrations > 70 pg/ml less than 12 h after vasopressors initiation were randomized to receive a single dose of adrecizumab or its placebo. With comparable adverse events between groups, the trial demonstrated the safety and tolerability of the drug. Although the trial was not designed for efficacy, patients receiving adrecizumab exhibited a more pronounced reduction in their SOFA score at day 7 and a comparable sepsis-support index (days alive without hemodynamic, respiratory or renal support) at day 30, while mortality at day 28 did not differ between groups (24 *vs*. 28% in the control group, p = 0.44). Further research is ongoing for the development of this drug during sepsis [159].

Given the deleterious role of vascular hyperpermeabilty and an association between circulating adrenomedullin levels and patient outcomes during cardiogenic shock, adrecizumab was also evaluated during this condition [164]. In the ACCOST-HH trial, a double-blind multicenter randomized trial, it did not provide any advantage on the primary endpoint of the number of days alive without cardiovascular organ support at day 30, nor on mortality (40 *vs* 40%, p = 0.98). Serious adverse events, however, were comparable between groups, further supporting the good tolerance of the drug [165].

#### FX06

FX06 is a drug containing the fibrin-derived peptide Bβ15–42, which stabilizes VE-cadherin-dependent inter-endothelial cell junctions [20, 166, 167]. It was first developed in murine myocardial I/R injury, reducing vascular leakage and tissue damage [168]. In a phase II trial conducted on 234 patients suffering from acute coronary syndrome, patients treated with FX06 exhibited a 58% decrease in their early necrotic core zone, although the primary endpoint of total infarct size at day 5 remained unaffected [169]. Importantly, adverse events were comparable between groups, indicating a high safety profile for the drug.

FX06 also reduced vascular leakage in several pre-clinical models of circulatory failure. In a pig model of cardiac arrest, i.v. FX06 decreased the need for fluid intake and improved the neurocognitive recovery of the animals [17]. Moreover, it reduced circulating levels of cytokines IL-1β, IL-6, TNF-α, IL-10, and MCP-1 in a mouse model of cecal-ligation-and-puncture-induced sepsis. It also reduced extravasation of i.v. injected fluorospheres and Evans blue in a mouse model of endotoxemia [20, 170]. Finally, during resuscitation from hemorrhagic shock in swine, FX06 attenuated markers of organ damage, and reduced circulating endotoxemia and inflammation [171, 172]. Nevertheless, FX06 yielded disappointing results in a phase II double-blind randomized study evaluating its efficacy during SARS-CoV-2-induced acute respiratory distress syndrome. In particular, it had no detectable effect on pulmonary edema [173].

Research is, however, currently ongoing in the setting of circulatory failure, particularly post-cardiac arrest shock, which would allow an early administration when compared to ARDS.

#### PCSK9 inhibitors

Proprotein Convertase Subtilisin/Kexin-9 (PCSK9) is a serine protease implicated in the homeostasis of low-density lipoprotein (LDL) receptors, key regulators of the inflammasome complex. During sepsis, PCSK9 is involved in its activation and the release of pro-inflammatory cytokines [174]. It has a more specific role on endothelial cell dysfunction, promoting ROS generation from the endothelium under inflammatory and abnormal shear stress conditions [175]. In a phase 2, double-blind randomized and controlled trial, the PCSK9 inhibitor evolocumab significantly decreased the intubation rate of patients with Sars-CoV-2-induced ARDS [176]. Studies are currently ongoing to characterize its role during human sepsis.

#### Modulators of angiopoietin/tie-2 pathway

Tie-2, a tyrosine kinase receptor expressed by endothelial cells, is a crucial regulator of endothelial permeability. Its main agonist, Angiopoietin-1 (Ang-1), promotes the vascular barrier function through cell–cell junction stabilization and strengthening of actin cytoskeleton, via activation of the Akt pathway [177]. In contrast, angiopoietin-2 (Ang-2), competing with Ang-1 for Tie-2 ligation, acts as a strong destabilizing agent of the endothelial barrier. Contained in Weibel-Palade bodies of endothelial cells, it is released at very early stages of endothelial cell activation [178]. Accordingly, Ang-2 levels directly related to pro-inflammatory cytokines concentrations during sepsis, and the Ang-1/2 ratio correlates with disease severity and outcome [179–181]. Hence, manipulating the angiopoietin/Tie-2 pathway thus also represents an attractive strategy to control vascular leakage during sepsis. Several drugs have been evaluated so far. Vasculotide, a synthetic Tie-2 agonist, has been shown to reduce cytokine response and, ultimately, dose-dependently improve survival in a fluid-resuscitated murine model of cecal ligation and puncture-induced polymicrobial sepsis [182]. Recombinant Ang-1 reduced vascular leakage and neutrophil infiltration in the lungs in a similar murine model of polymicrobial, abdominal sepsis [183]. Finally, an anti-Ang-2 antibody with Tie-2 agonist properties also demonstrated protective effects on the vasculature and improved survival in three other different murine models of sepsis [184]. A phase 2a study is currently recruiting to evaluate the safety and tolerability of the synthetic Tie-2 agonist AV-001 in humans with pneumonia (NCT05123755).

#### Other strategies

Many other strategies aiming at correcting dysregulated inflammation are currently investigated during the different forms of circulatory failure (mostly during sepsis), which may indirectly help controlling vascular leakage [146, 185]. Moreover, better understanding of patients heterogeneity regarding inflammatory phenotypes may help reappraising treatments that failed demonstrating their efficacy in humans, despite encouraging results in pre-clinical studies [186, 187].

## Conclusion

Inflammation-induced vascular leakage is a major contributor to organ dysfunction during circulatory failure and is thought to crucially impact patients’ outcome. Despite promising pre-clinical findings, none of the tested drugs translated to routine clinical care. Vascular hyperpermeability during shock, thus, remains an area of research to better characterize its mechanisms in humans and consecutively develop new treatments. Better characterization of the individual patient’s phenotype associated with vascular leakage is also mandatory to further evaluate these new treatments.

## Ackowledgments

We thank Obaid Alzaabi, for his participation in manuscript preparation, and France Maloumian for her help in the figures.

## Availability and data materials

Not applicable.

## Data Availability

All original data provided in this review will be shared beginning with publication with no end date. These data will be available to researchers to who provide a methodologically sound proposal for the purposes of achieving specific aims outlined in that proposal. Proposals should be directed to the corresponding author via email: nicolas.brechot@aphp.fr and will be reviewed by the senior authors of the study.

## Abbreviations

DAMPS: Damage-associated molecular patterns
EC: Endothelial cell
FGF: Fibroblast growth factors
HUVEC: Human umbilical vein endothelial cells
IL: Interleukin
KLF: Krüppel-like family of transcription factors
LPS: Lipopolysaccharide
L-HMVEC: Lung human microvascular endothelial cell
PAMPS: Pathogen associated molecular patterns
PCSK9: Proprotein convertase subtilisin/kexin-9
PECAM: Platelet endothelial cell adhesion molecule
PRR: Pattern-recognition receptors
ROS: Reactive oxygen species
SOD: Superoxide dismutase
TLR: Toll-like receptor
TNF-Α: Tumor necrosis factor-alpha
VE-CADHERIN: Vascular endothelial-cadherin
VEGF: Vascular endothelial growth factor
VEGFR: Vegf receptor
